# Channel Characteristic Aware Privacy Protection Mechanism in WBAN

**DOI:** 10.3390/s18082403

**Published:** 2018-07-24

**Authors:** Puning Zhang, Jie Ma

**Affiliations:** Department of Communication and Information Engineering, Chongqing University of Posts and Telecommunications, Chongqing 400065, China; michaelmajie@foxmail.com

**Keywords:** wireless body area network, privacy protection, node authentication, information encryption

## Abstract

Advances of information and communication technologies in medical areas have led to the emergence of wireless body area network (WBAN). The high accessibility of media in WBAN can easily lead to the malicious tapping or tampering attacks, which may steal privacy data or inject wrong data. However, existing privacy protection mechanisms in WBAN depend on the third-party key management system and have a complex key exchange process. To enhance user privacy at a low cost and with high flexibility, a channel characteristic aware privacy protection mechanism is proposed for WBAN. In the proposed mechanism, the similarity of RSS is measured to authenticate nodes. The key extraction technique can reduce the cost of the key distribution process. Due to the half duplex communication mode of sensors, the biased random sequences are extracted from the RSS of sensor nodes and coordinator. To reduce the inconsistency, we propose the n-dimension quantification and fuzzy extraction, which can quickly encrypt the transmission information and effectively identify malicious nodes. Simulation results show that the proposed mechanism can effectively protect user privacy against tampering and eavesdropping attacks.

## 1. Introduction

Wireless Body Area Network (WBAN) employing wireless media to provide data transmission services is the core component of many telemedicine applications, such as personalized medicine and home-based mobile health [1]. It replaces complex and wired healthcare equipment to continuously monitor vital information without hampering user movements.

WBAN usually involves the star network topology like the centralized topology of peer to peer network [2]. All sensor communicate directly with the sink node. However, due to the high accessibility of wireless media and the lack of tamper resistant hardware on sensors, WBAN is vulnerable to malicious attacks [3]. and dangers hide in the communication security of WBAN [4]. The monitored physiological data of patients inevitably are prone to attacks, such as the eavesdropping attack and tampering attack [5]. In particular, the physiological data transmitted in WBAN have high privacy requirements. Once the monitoring process is eavesdropped or the physiological data are tampered, patient privacy is leaked and human health faces grave threats. Therefore, the privacy protection has become a major research problem of WBAN in recent years.

At present, the solutions to the privacy leaks in WBAN mainly include the node authentication and information encryption. WBAN is usually composed of the coordinator and sensor nodes, where the coordinator is the so-called sink which serves as the gateway to another WBAN, a trust center or an access coordinator [6]. The node authentication is the legitimacy determination of node identity through communications between sensors and coordinator.

The traditional node authentication exploits key sharing strategies to authenticate the node identity. However, malicious attackers can steal the key through wiretapping key exchange. Information encryption is another technology exploiting mathematical or physical means to avoid privacy leaks in the transmission process. The basic requirement of information encryption is the key generation and the most common key generation method is the asymmetric key. In this way, a third-party key management system is authorized to distribute asymmetric keys to nodes through the secure channel, by which the privacy data can be protected [7,8,9]. Sensors in WBAN have many resource constraints, such as computing, transmission and power. Due to the limitations of hardware and software, sensors cannot afford techniques commonly employed in traditional networks to establish the secure communications [10]. Specifically, the battery resources of WBAN sensors should be fully exploited [11]. Security solutions can be excessively expensive in terms of power consumption. In essence, this is rooted in how such mechanisms are designed [12]. However, conventional encryption technology relying on the third-party key management system incurs heavy computation cost. Therefore, privacy leaks in WBAN have to be solved urgently and skillfully.

In this paper, the independent key establishment scheme without third-party key system is proposed to prevent privacy leaks. The main contributions of this research work are as follows.

The main contributions of the paper are summarized as follows:

- the channel characteristic similarity estimation mechanism is proposed to authenticate the legitimate sensors, therefore malicious attackers cannot pass the authentication due to the channel characteristic difference between them and normal sensors.
- by utilizing the RSS between sensors and coordinator, extraction key is adopted to realize the node authentication and information encryption. Because of the instantaneous change of RSS in the wireless channel, the extraction key is dynamic and the information varies over time so that the privacy data can be protected.
- the inconsistency removal multidimensional quantification and key unification mechanisms are adopted to ensure the low bit inconsistency rate and high key generation rate.

The remainder of this paper is organized as follows. In Section 2, an overview of related work is given. In Section 3, the network model of WBANs is proposed. In Section 4, the channel characteristic aware privacy protection mechanism is introduced. In Section 5, numerical results are presented and analyzed. Finally, the conclusion is given in Section 6.

## 2. Related Works

In this section, we review existing node authentication and information encryption schemes.

The work in [8] proposed a mutual authentication scheme for WBAN, which realized the mutual authentication of legitimate nodes and encrypted the transmission data using the public key. The proposed technique employed asymmetric encryption. However, while really effective for implementing sophisticated and robust key exchange and verification tasks, it is not suitable in terms of the battery or computing capabilities [13]. Energy itself, as an entity, should be protected [14]. Thus, it is inapplicable to resource-constrained WBAN. The authors in [9] put forward a security system for WBAN, which authenticated the legitimate nodes with the hash value combined with node identification and random numbers. Meanwhile, the proposed security system encrypted and decrypted the data with a shared symmetric key. However, with the development of computing technology such as the quantum computers, the ability of attackers is significantly improved, which seriously threatens such key sharing-based privacy protection methods. Therefore, the physical layer security methods without relying on the key sharing-based privacy protection were proposed to establish keys independently based on the physical layer information [15,16,17,18,19,20,21,22,23,24,25,26,27]. The physical layer security method in [15] was proposed based on information security theory and provided information encryption keys for the upper layer [16]. The physical layer information for key generation included the signal arrival phase [17], angle [18], level cross point, signal envelope, channel pulse [19] and reception signal intensity. The work in [20] employed sensor nodes to collect biological characteristics such as cardiac electrical signals for node authentication and key generation. However, these physiological data are prone to noise and other environment factors and cannot ensure consistency. The work in [25,26] proposed HRUBE and ARUBE schemes, which adopted multiple interval quantification, KLT transformation, rank order and gray code error correction for Received Signal Strength (RSS) to achieve the independent key generation. The bit quantization methods could be divided into single bit quantization and multi bit quantization [27]. When key was extracted from different environments such as rotating, sitting, wavering and walking, although the bit inconsistency rate of single bit quantization was low, the key generation rate of single bit quantization was unsatisfactory. Multi bit quantization can improve the key generation rate, but its bit inconsistency rate is high. Therefore, the above technologies cannot provide the low bit inconsistency rate and high key generation rate.

In this paper, to effectively guarantee the applicability and reliability of node authentication and information encryption in WBAN, and to meet the bit inconsistency and key generation rate requirements, a channel characteristic aware privacy protection mechanism is proposed. Simulation results show that the proposed privacy protection mechanism only relies on the physical layer characteristics, which can not only secure the secure communication, but also achieve low bit inconsistency rate and high key generation rate.

## 3. Network Model

In WBAN, each sensor node collects and sends physiological data to a coordinator through wireless channel. In particular, the computing power, memory space and energy supply of sensors are limited. The coordinator gathering the uploaded data can be a smartphone or a tablet computer, then it forwards data to the trusted local or remote data processing system, like an emergency service center. The coordinator close to the physical locations of all sensors is placed near the body, and the distances between the coordinator and sensors are mostly less than one meter.

**Figure 1:**
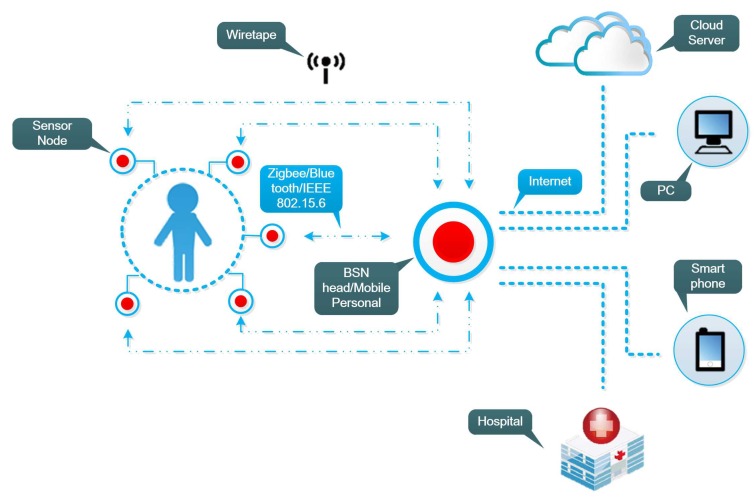
WBAN Network Model.

However, in actual application scenarios, malicious attackers randomly appear around the patient or key management center, and try to disguise themselves as legitimate sensors to attack or wiretap the key distribution process. As shown in Figure 1, attackers can forge physical addresses, wiretap wireless channels and inject false data. Compared to legitimate sensors, their physical locations are far away from the wearer. Therefore, the wireless channel environment of malicious attackers presents notable difference with that of legitimate nodes.

The work in [28] affirmed that the wireless channel characteristics can be used as random signal sources, which have spatiotemporal uniqueness, randomness and reciprocity. Therefore the RSS, as the statistic channel parameter between the transmitter and receiver, can be quantified to generate keys. Because of its uniqueness and randomness, malicious attackers cannot obtain the actual RSS values from different locations. In the data communication process of WBAN, there exist some cases such as body masking, reflection, diffraction and absorption, which lead to channel fading. For a given slot *t*, the RSS value of channel (*S*, *C*) between the coordinator *C* and sensor *S* is defined as:

(1) $${\hat{Y}}_{s}\left(t\right)=Y_{s}\left(t\right)+W_{s}\left(t\right)$$

where $Y_{s}\left(t\right)$ is the channel gain in the slot *t*, and $W_{s}\left(t\right)$ is the observed noise gain.

Therefore, a sensor can obtain the observed value of RSS. The privacy protection mechanism proposed in this paper can use observed RSS values to carry out the node authentication and key generation for WBAN. First, the legitimate sensors have almost the same channel quality and RSS, which realize the fast sensor authentication. Second, the key pairs are randomly extracted from the RSS between nodes, which can prevent attackers from tampering and eavesdropping. The whole process does not involve the third-party key management system, complex settings, additional hardware or other changes, which is practical for WBAN.

## 4. Channel Characteristic Aware Privacy Protection Mechanism

Node authentication and information encryption are designed in this section. The node authentication algorithm exploits the characteristics of wireless channel to authenticate legitimate nodes based on the correlation coefficient. The information encryption algorithm should meet three requirements: (1) The key of two node has the same sequence; (2) The key of two node has the appropriate length (128 bit to 512 bit); (3) The key has statistical randomness to encrypt the sending information. The proposed mechanism can prevent malicious attackers from wiretapping or impersonating legitimate nodes to forge data, and employs the observed RSS values to extract keys to avoid privacy leaks caused by key sharing.

### 4.1. Node Authentication

When sensors communicate with the coordinator, they obtain the observed RSS values by sending the detection packet. The RSS value can reflect the channel quality, which varies with the change of environment and body movements. The channel environment around the human body is complex and the RSS is influenced by many factors such as multipath and occlusion. Besides, the RSS may also be affected by body parts and their positions. However, the positions of attackers are much different from those of legitimate nodes and the attackers may only be influenced by environment factors such as multipath. Therefore, the RSS of a malicious attacker is not related to that of a legitimate node, and the attacker also cannot predict the observed RSS value of a legitimate node.

The RSS can reflect the approximate degree between environments of two nodes [29]. Let Y(d) and $Y(d_{0})$ denote the received signal intensity of arbitrary distance *d* and reference distance $d_{0}$ to the coordinator, then the RSS value of the coordinator receiving the signals from sensors can be specifically expressed as:

(2) $$\begin{array}{cc} Y(d) & =Y\left(d_{0}\right)-10\beta lg\left(\frac{d}{d_{0}}\right)+X\\ & =10\mathrm{lg}\left(\frac{H_{s}G_{s}G_{c}\lambda^{2}}{16\pi^{2}{d_{0}}^{2}f}\right)-10\beta \mathrm{lg}\left(\frac{d}{d_{0}}\right)+X \end{array}$$

where $H_{s}$ is the transmission power of a sensor, $G_{s}$ is the antenna gain of a sensor, $G_{c}$ is the antenna gain of the coordinator, *f* is the system loss factor, λ is the wavelength of the wireless signal, β is the path loss factor, *X* is a normal random variable and $X∼N(0,\sigma^{2})$.

According to Equation (2), because sensors are in the similar environment, the environment parameters, such as the system loss and distance to the coordinator, are similar, and the RSS values between sensors and the coordinator are correlated, which will be proven below. While the environment of malicious nodes is different from that of legitimate nodes, and the distances between the coordinator and malicious nodes are larger than those between the coordinator and legitimate nodes. Therefore, the RSS values of malicious nodes are barely correlated with those of legitimate nodes.

In other words, the legitimate nodes have their own characteristics. For example, their locations, distances to coordinator and channel quality are similar, thus their RSS values present strong correlations, which can be exploited to calculate the RSS correlation to authenticate nodes.

The legitimate nodes transmit the detection packets to obtain the observed RSS values. Although the locations of legitimate nodes are different, their relative distances are much closer. Similar to the channel quality of the coordinator, the observed RSS values of sensors have certain correlations. When the coordinator authenticates sensors, the correlation coefficient between the RSS values can be used.

The coordinator broadcasts packets to initiate the RSS measurement value with the detection packets. Assume the observed RSS value between sensor $s_{1}$ and coordinator is $Y_{s_{1}}$, and the observed RSS value between sensor $s_{2}$ and coordinator is $Y_{s_{2}}$. Although the locations of the two sensors are different, their distances to the coordinator are close. Therefore, their observed RSS values are similar. *c* is the observation coefficient, which improves the observed RSS value. The similarity between the observed RSS value of $s_{1}$ and the improved RSS value of $s_{2}$ can be expressed as

(3) $$\overset{\_}{\underset{\left(Y_{s_{1}},Y_{s_{2}}\right)}{\varepsilon^{2}}}=\int_{-\infty }^{\infty }{\left[Y_{s_{1}}\left(t\right)-cY_{s_{2}}\left(t\right)\right]}^{2}dt$$

where $\overset{\_}{\underset{\left(Y_{s_{1}},Y_{s_{2}}\right)}{\varepsilon^{2}}}$ is the mean square error between the observed RSS values of $s_{1}$ and $s_{2}$. Because the observed RSS values of $s_{1}$ and $s_{2}$ have a certain similarity, when $\frac{{\bar{d\varepsilon^{2}}}_{\left(Y_{s_{1}},Y_{s_{2}}\right)}}{dc}=0$, $\overset{\_}{\underset{\left(Y_{s_{1}},Y_{s_{2}}\right)}{\varepsilon^{2}}}$ is the minimum, namely

(4) $$c_{12}=\frac{\int_{-\infty }^{\infty }Y_{s1}\left(t\right)Y_{s2}\left(t\right)dt}{\int_{-\infty }^{\infty }{Y_{2}}^{2}\left(t\right)dt}$$

(5) $$\overset{\_}{\underset{\left(Y_{s},Y_{c}\right)}{\varepsilon^{2}}}=\int_{-\infty }^{\infty }{\left[Y_{s1}\left(t\right)-Y_{s2}\left(t\right)\frac{\int_{-\infty }^{\infty }Y_{s1}\left(t\right)Y_{s2}\left(t\right)dt}{\int_{-\infty }^{\infty }{Y_{s2}}^{2}\left(t\right)dt}\right]}^{2}dt$$

The correlation coefficient $\rho_{\left(Y_{s1},Y_{s2}\right)}$ can be expressed by

(6) $$\rho_{\left(Y_{s},Y_{c}\right)}=\frac{\int_{-\infty }^{\infty }Y_{s}\left(t\right)Y_{s2}\left(t\right)dt}{{\left[\int_{-\infty }^{\infty }{Y_{s1}}^{2}\left(t\right)dt\int_{-\infty }^{\infty }{Y_{s2}}^{2}\left(t\right)dt\right]}^{\frac{1}{2}}}$$

If $\rho_{\left(Y_{s1},Y_{s2}\right)}=1$, $\overset{\_}{\underset{\left(Y_{s1},Y_{s2}\right)}{\varepsilon^{2}}}=0$, and $Y_{s_{1}}$ is the same as $Y_{s_{2}}$. If $\rho_{\left(Y_{s1},Y_{s2}\right)}$ is approaching 0, $\overset{\_}{\underset{\left(Y_{s1},Y_{s2}\right)}{\varepsilon^{2}}}$ is reaching its maximum value, and they are orthogonal to each other.

According to Equation (2), the coordinator’s observations for two sensors are as follows:

(7) $$Y{\left(d\right)}_{1}=10\mathrm{lg}\left(\frac{H_{s1}G_{s1}G_{c}\lambda^{2}}{16\pi^{2}{d_{0}}^{2}f}\right)-10\beta_{1}\mathrm{lg}\left(\frac{d_{1}}{d_{0}}\right)+X_{1}$$

(8) $$Y{\left(d\right)}_{2}=10\mathrm{lg}\left(\frac{H_{s_{2}}G_{s_{2}}G_{c}\lambda^{2}}{16\pi^{2}{d_{0}}^{2}f_{2}}\right)-10\beta_{2}\mathrm{lg}\left(\frac{d_{2}}{d_{0}}\right)+X_{2}$$

In order to analyze the correlation between $Y{\left(d\right)}_{1}$ and $Y{\left(d\right)}_{2}$, the difference between the Formulas (7) and (8) can be expressed as:

(9) $$\begin{array}{c} Y{\left(d\right)}_{1}\left|{}_{\mathrm{dBm}}\right.-Y{\left(d\right)}_{2}\left|{}_{\mathrm{dBm}}\right.=10\mathrm{lg}\left(\frac{H_{s1}G_{s1}f_{2}}{H_{s2}G_{s2}f_{1}}\right)-10\mathrm{lg}{\left(\frac{d_{1}}{d_{2}}\right)}^{\beta_{1}}{\left(\frac{d_{0}}{d_{2}}\right)}^{\Delta \beta }+{\left[X_{1}-X_{2}\right]}_{dB} \end{array}$$

where $\Delta \beta =\beta_{2}-\beta_{1}$. As shown in Equation (9), because two legitimate nodes are close to each other, the difference of the distance between them and the coordinator tends to 1. Due to the similar environmental factors, the transmission power rate *H*, antenna gain *G* and loss factor *f* of the system tend to be constant; the path loss factor β and random variable *X* tend to be equal; Δβ goes to 0, the observed the RSS value is close to a constant, and the RSS difference is also close to a constant. However, the environment of malicious nodes varies greatly, there are no evident correlations between their RSS and those of legitimate sensors. Therefore, RSS between legitimate nodes has a certain correlation, and the correlation coefficient among legitimate nodes is close to 1. While the RSS correlation between malicious nodes and legitimate nodes is weak and the correlation coefficient is close to 0.

The notations used in the system are summarized in Table 1.

Therefore, the off-line RSS samples $Y=(Y_{1},Y_{2},\ldots ,Y_{n})$ of *n* nodes are exploited to calculate the similarity between them, and the maximum mean square error $\overset{\_}{\underset{\max}{\varepsilon^{2}}}$ is defined as the similarity threshold. In the actual authentication process, when $\varepsilon^{2}$ of a node is greater than similarity threshold $\overset{\_}{\underset{\max}{\varepsilon^{2}}}$, the node is identified as a malicious node. On the contrary, when $\varepsilon^{2}$ of a node is less than or equals similarity threshold $\overset{\_}{\underset{\max}{\varepsilon^{2}}}$, the node is legitimate.

### 4.2. Encrypting Information

After the authentication between the coordinator and legitimate sensors, the system sends the communication information. A new key establishment algorithm based on channel quality is proposed to symmetrically encrypt and transmit the information. In this algorithm, every sensor extracts effective key independently without key distribution process, which makes it impossible for a malicious attacker to acquire keys by eavesdropping.

In order to extract the effective key, the coordinator and the sensor node, as a pair of transceiver nodes, obtain the random sequences separately by quantifying the RSS values. However, due to the half duplex communication mode of sensors, RSS can only be measured unidirectionally at the same time. Therefore the random sequences extracted by the sensor and coordinator are biased. Once the pair of transceiver nodes extract the random sequences from the measured values, the inconsistent random sequences have to be corrected to obtain the secret key between the coordinator and sensor.

Therefore, an efficient key extraction method based on multidimensional quantification is proposed to correct the inconsistent bits in the two random sequences of the sensor and coordinator. Then increasing the generation length of secret keys can reduce the bit inconsistency rate and rise the key generation rate. First, we quantify the RSS with the inconsistency removal method and increase the sequence length with the n-dimensional quantifier. Finally, the random sequence obtained by the n-dimension quantifier is transformed into the same secret key between the coordinator and sensor through the fuzzy key unification method.

#### 4.2.1. The Inconsistency Removal Method

Due to the half duplex communication mode, the traditional quantification of RSS values generates the inconsistent sequences. Specifically, traditional quantification schemes are bounded by the average value to quantify RSS, which is 1 (or 0) higher than the average value, and 0 (or 1) lower than the average value. However, the RSS values near the mean value measured by sensor node and coordinator easily shift. And the results of traditional quantification methods are inconsistent. To reduce the inconsistency, a quantification method is proposed in this paper with upper bound $q^{+}$ and lower bound $q^{-}$ to quantify RSS. And then the RSS values above $q^{+}$ or less than $q^{-}$ are retained and others are discard. Therefore, RSS values having great differences with the average value are retained and RSS values that cause the inconsistency of sequences are discarded. The upper and lower bounds are set as:

(10) $$q^{+}=v-\alpha \times d$$

(11) $$q^{-}=v-\alpha \times d$$

where α is the wave factor, 0<α<1, *v* is the average RSS value and *d* is the standard deviation of the RSS value. The removal function L(x) is defined as follows:

(12) $$L\left(x\right)=\left\{\begin{array}{c} 1\;x>q_{}^{+}\\ 0\;x<q_{}^{-}\; \end{array}\right.$$

**Figure 2:**
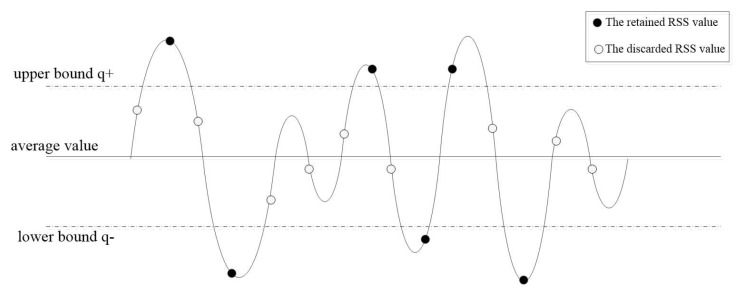
RSS filter quantizer.

As shown in Figure 2, the RSS values greater than $q^{+}$ and less than $q^{-}$ are 1 and 0, respectively. The values between $q^{+}$ and $q^{-}$ are discarded to effectively eliminate the inconsistency between sequences of the sensor and coordinator.

Because there are too many RSS values to be collected, the average values of RSS in different time regions are diverse. To accurately quantify RSS, their values are divided into blocks and the block length is *b*. After the segmentation and quantification, the generated sequences are spliced to the final sequence. The sequence is used as input to the *n*-dimensional quantification process.

#### 4.2.2. The *n*-Dimensional Quantification

Because the inconsistency of RSS values is removed, the sequence generated is smaller. An  efficient *n*-dimensional quantification method is proposed to improve the key generation rate without increasing the bit inconsistency.

The *n*-dimensional vector is established to sample the sequences. The input values of the *n*-dimensional quantification scheme are $Y=\left\{y_{1},y_{2},\ldots ,y_{d}\right\},y_{i}\in Z$ which are the output of the inconsistency removal method, where *d* is the length of sequence *Y*, and then the *n*-dimensional vector is established as follows:

(13) $$<y_{i},y_{(i+\Delta_{1})\;\mathrm{mod}\;d},\ldots ,y_{(i+\Delta_{1}+\ldots +\Delta_{n-1})\;\mathrm{mod}\;d}>$$

where $\Delta =\{\Delta_{1},\Delta_{2},\ldots ,\Delta_{n-1}\}$ represents the sampling interval. The $\Delta_{j}$ is the interval of time slot between *j* and j+1. For a random sequence, bits of corresponding positions are selected according to each element in *Y*, and then we rearrange them to generate the new random sequence. After multidimension sampling, the output value can be expressed as follows:

(14) $$Q_{N}\left(y_{i}\right)=R\left(y_{i}\right)R\left(y_{(i+\Delta_{1})\;\mathrm{mod}\;d}\right)\ldots R\left(y_{(i+\Delta_{1}+\ldots +\Delta_{n-1})\;\mathrm{mod}\;d}\right)$$

where R(x) is the corresponding quantification function.

The whole process can be regarded as an *n*-dimensional quantification with the random sequence as the input and the random sequence results as the output. The quantification method takes $y_{i}$ as the standard to quantify values in the random sequence until the *n*-th value, and then it rearranges the values in the sequence to generate the new random sequence. The quantification outputs a new random sequence whose length is *n* and the bit generation rate of the *n*-dimensional quantification is 1:*n*. The pseudo code of the algorithm is shown in Algorithm 1. A two-dimensional quantification is taken as an example to illustrate the *n*-dimensional quantification process. The quantifier searches the second component $y_{\left(i+\Delta_{1}\right)}$ when $y_{i}$ is input into the system, and then outputs sequence $R\left(y_{i}\right)R\left(y_{(i+\Delta_{1})\;\mathrm{mod}\;d}\right)$. The quantification function is shown as follows:

(15) $$Q\left(y_{i}\right)=\left\{\begin{array}{c} 00\;y_{i}<q^{-},y_{(i+\Delta_{1})\;\mathrm{mod}\;d}<q^{-}\\ 10\;y_{i}>q^{+},y_{(i+\Delta_{1})\;\mathrm{mod}\;d}<q^{-}\\ 11\;y_{i}>q^{+},y_{(i+\Delta_{1})\;\mathrm{mod}\;d}>q^{+}\\ 01\;y_{i}<q^{-},y_{(i+\Delta_{1})\;\mathrm{mod}\;d}>q^{+} \end{array}\right.\;$$

**Algorithm 1**
*n*-dimensional quantification algorithm
1:$\Delta_{t}\leftarrow 0$;2:**for** i=1 to *d* **do**3: $Q_{N}\left(y_{i}\right)\leftarrow R\left(y_{i}\right)$;4: $\Delta_{t}\leftarrow 0$;5: **for** j=1 to N−1 **do**6:  $\Delta_{t}\leftarrow \Delta_{t}+\Delta_{j}$;7:  $Q_{N}\left(y_{i}\right)\leftarrow strcat\left(Q_{N}\left(y_{i}\right),R\left(y_{(i+\Delta_{t})modd}\right)\right)$;8: **end for**9:**end for**10:$B\leftarrow Q_{N}\left(y_{1}\right)Q_{N}\left(y_{2}\right)\cdots Q_{N}\left(y_{d}\right)$;11:END


#### 4.2.3. The Establishment of Consistent Key

**Figure 3:**
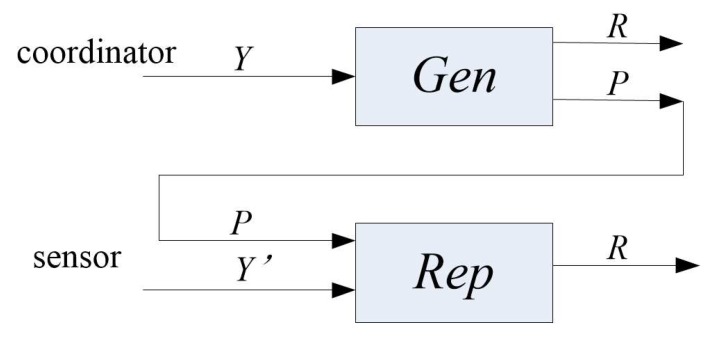
The fuzzy extractor.

The sequences generated by the sensor and coordinator are processed by the inconsistency removal and *n*-dimensional quantification methods. To generate effective keys from the sequences of sensor node and coordinator for information encryption, the fuzzy key unification method is introduced, which corrects errors according to confusion and diffusion properties and selects the appropriate hamming distance to map to the same random sequence. It not only allows two similar random sequences to map to the same sequence, but also converts a low entropy sequence to a random sequence. The fuzzy extractor is shown in Figure 3. The BCH code [30] in the fuzzy extractor is selected to correct the errors and the SHA-1 function is used to enhance the randomness of the sequence. Therefore, it solves the low randomness and inconsistency of secret keys.

The fuzzy extractor (*M*,*l*,*t*) contains the generation and regeneration process, namely Gen process and Rep process in this method, where *M* is a string of input sequences, *l* is the length of key *R*, and *t* indicates that the Hamming distance of two sequences can generate the same random sequence without exceeding *t*.

In the generation process, the coordinator inputs Y∈M to Gen and then it outputs random sequence *R* and public information *P*. The coordinator shares public information *P* with a sensor to generate key R in the second step, and it can be expressed by Gen(Y)→(R,P).

In the process of regeneration, the sensor inputs *P* to Rep and it will output *R* when hamming distance $dis(Y,Y^{\prime })\leq t$, which can be expressed as $Rep(Y^{\prime },P)\to R$.

Therefore, sequence *Y* generated by coordinator after the inconsistency removal and *n*-dimensional quantification is input to Gen, and then the public information *P* and secret key *R* are generated. The coordinator sends *P* to the sensor node, and the sensor node inputs it to Rep together with random sequence *Y* to generate the same secret key *R*. The sensor node encrypts the information with secret key *R* and sends it to coordinator. And then the coordinator receives the information to decrypt the information.

## 5. Numerical Results

In order to analyze the performance of channel characteristic aware privacy protection mechanism for WBAN. The number of packets per second is 1. The packet payload is assumed to be 1020 bits. We carry out experiments in different settings, mainly aiming at the validity of authentication, the length of key extraction and inconsistency rate. Simulation includes various factors that affect the performance, such as the postures of sitting and rotating, sitting, sitting and rolling, and walking. Note that because the tester is in the motion mode, the distance between the coordinator and the sensor node is not strictly fixed, which is about 10 feet. The sampling time of the received signal intensity is 20 ms. BANA [31] and MASK-BAN [32] are compared with the proposed mechanism under the same conditions. BANA extracts the key quantified by RSS directly, and uses clustering analysis to authenticate, whereas MASK-BAN uses the multi hop method to authenticate legitimate nodes, and builds up the maximum flow problem with relay nodes to extract key.

**Figure 4:**
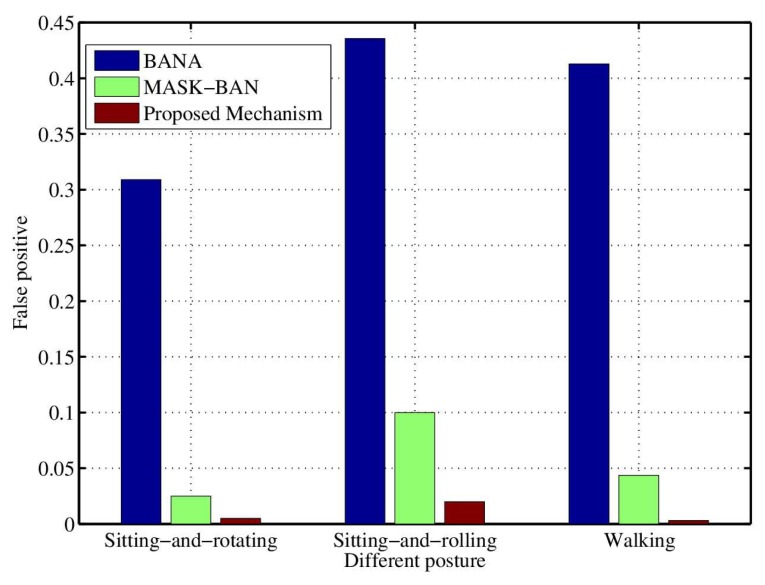
The influence of various actions on the false probability of authentication.

Figure 4 shows the false positive, namely the probability of authentication error, which is the probability of authenticating a malicious attacker into a legitimate node. The graph shows that the scheme is better than the other two mechanisms. The BANA mechanism adopts cluster analysis, while the number of nodes in WBAN is small, and the observed data are few, so the authentication is inaccurate.

**Figure 5:**
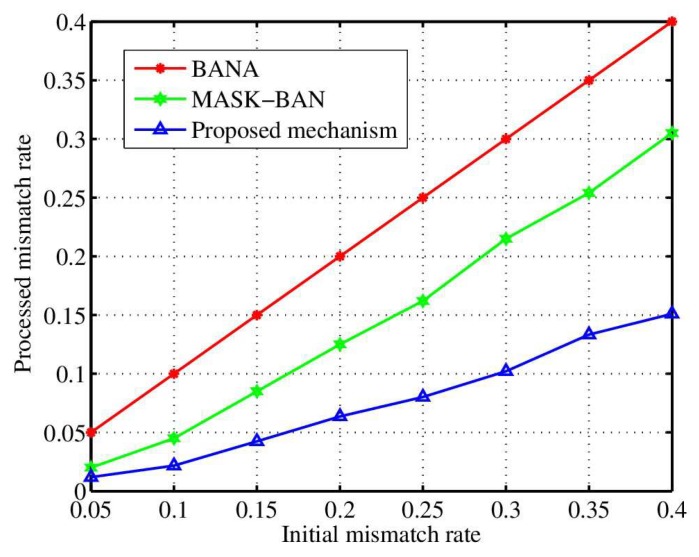
The influence of inconsistent initial bits on the inconsistent key rate.

Figure 5 shows the inconsistent key rate in the case of inconsistent initial bits. The coordinator uses the fuzzy extractor to make the same sequences of sensor node and coordinator as the effective keys for information encryption and quantifies the RSS by the *n*-dimension method. The coordinator uses the *n*-dimensional quantization sequence as the Gen input. And then public information *P* is created by BCH (23, 12) and random key *R* with strong randomness is generated with the SHA-1 function. Then the coordinator sends public information *P* to the sensor, and the sensor restores its sequence $Y^{\prime }$ to *Y* according to public information *P*. And then the sensor generates the same strong random secret key *R* with the SHA-1 function. In the simulation, the length of the original BCH (23, 12) code is 12 bits, the length of error correcting code *P* is 11 bits, and the minimum code distance is 7 bits which can correct 3 error bits. Therefore, the key generated by this method has very low inconsistent key rate.

**Figure 6:**
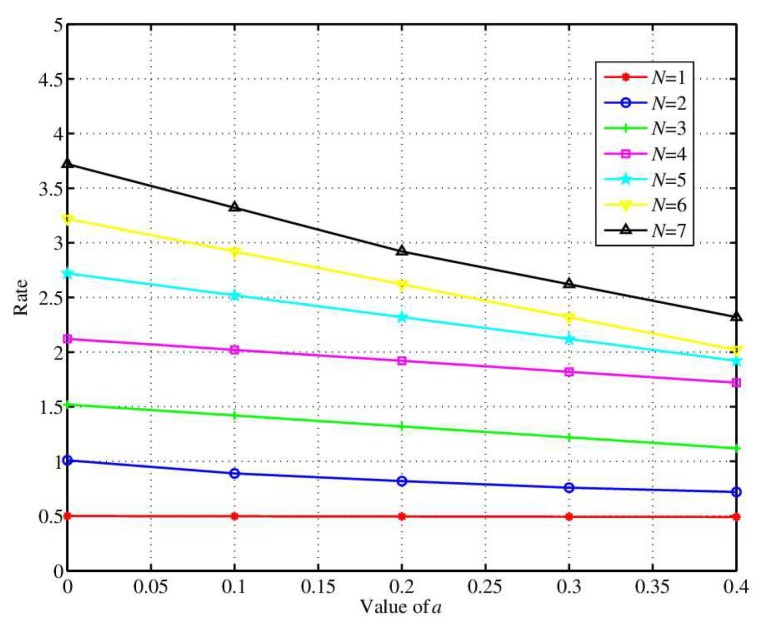
The change of bit generation rate with α different dimensions.

**Figure 7:**
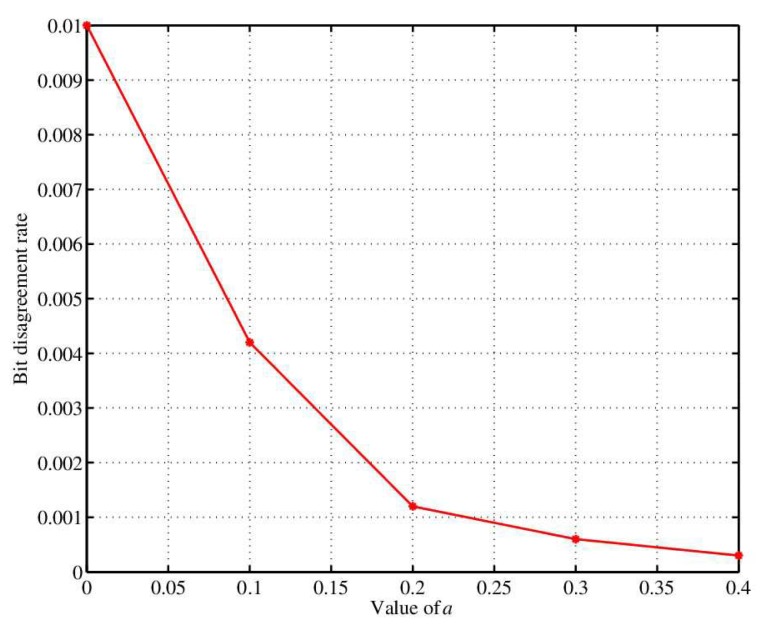
The variation of bit inconsistency with α.

When the sensor and coordinator quantify the channel characteristics and RSS by using the inconsistency removal method separately, parameter α is introduced. When α is large and the difference between $q^{+}$ and $q^{-}$ is far away, the retained RSS value is far away from the average line. The experimental results of the effect of α on key generation rate and bit inconsistency rate are shown in Figure 6 and Figure 7, respectively. Obviously, the higher dimension signifies the smaller key generation rate and the higher bit inconsistency rate. Therefore, the key generation rate and bit inconsistency rate should be considered simultaneously in practical applications and parameter α is set to 0.2.

In this paper, the *n*-dimensional quantization is used to increase the key generation rate without increasing bit inconsistency rate, where *n* is the quantification degree. The larger *n* signifies the higher bit generation rate and affects the randomness of the generated keys. In order to determine the impact on the key, the key generated by different dimensions is evaluated with the 9 test methods of NIST. For each test, if the value is greater than 0.01, the test is pass. The assessment results are shown in Table 2. When n≥8, bits are reused to generate keys and the independence between each bit is reduced. When the NIST measurement FFT value is 0, which fails to pass the randomness test. Therefore, 7 dimensional quantification is an upper bound for ensuring the randomness of key generation.

**Figure 8:**
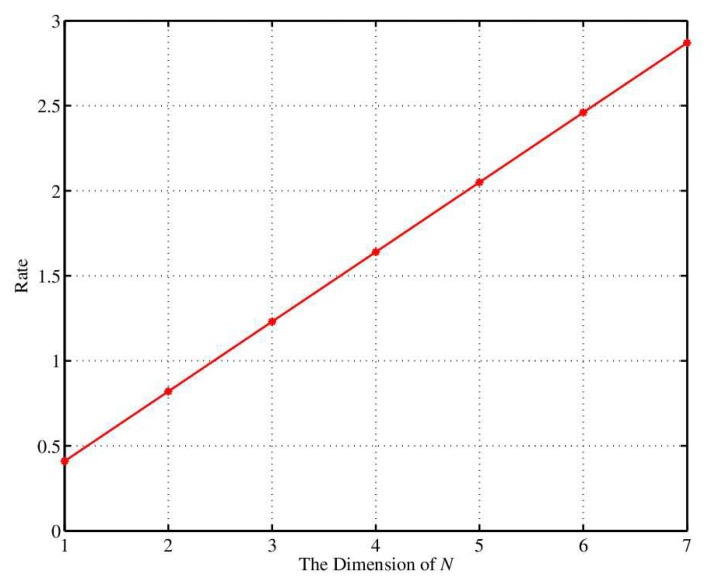
The change in the bit generation rate with the increase of dimension.

Figure 8 is the bit generation rate of the *n*-dimensional quantification. When the parameter is fixed to α=0.2, $\Delta_{1}=\Delta_{2}=\cdots =\Delta_{n-1}=60$, the bit generation rate increases with the growing dimension. When the dimension is 7, the key generation rate can reach 286%.

**Figure 9:**
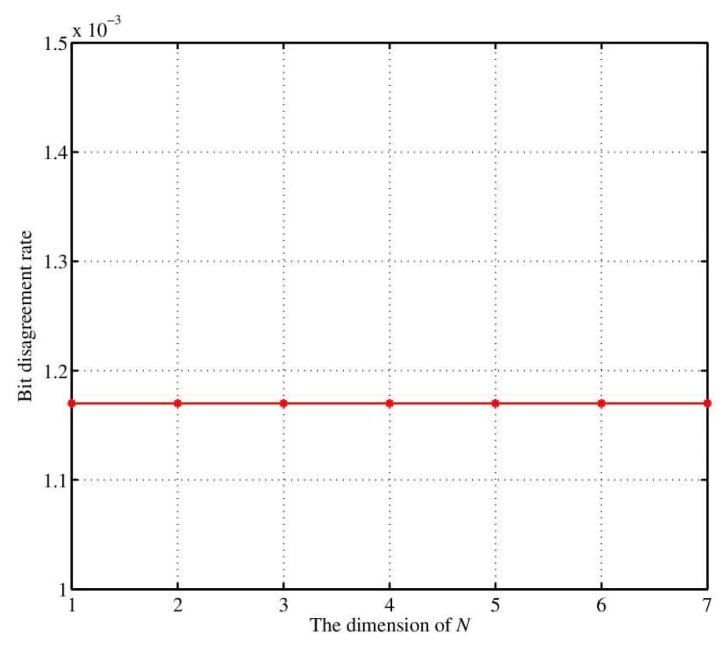
The variation of the bit disagreement rate with the dimension.

Figure 9 shows the bit inconsistency rate of the *n*-dimensional quantification method. It can be seen that the sequence already have relatively low bit quantification rate before the fuzzy extraction. Thus it provides a better input sequence for the fuzzy extractor. Because the bit inconsistency rate before the fuzzy extraction is very low, the fuzzy extractor can correctly output the same secret key.

**Figure 10:**
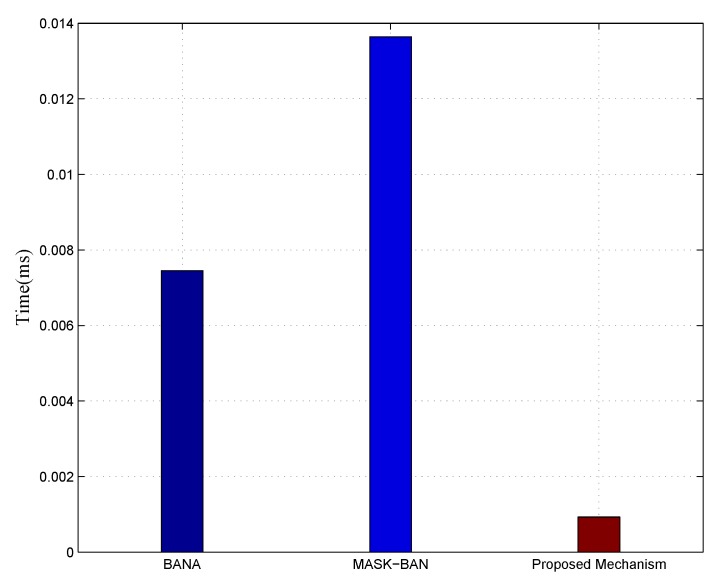
Complexity benchmarking based on execution time.

The average time of executing node authentication, key extraction and information encryption is calculated to reflect the complexity. The execution time that BANA and MASK-BAN consume are 74.5 × 10${}^{-4}$ ms and 136.5 × 10${}^{-3}$ ms, respectively. On the other hand, for the same purposes, the channel characteristic aware privacy protection mechanism for WBAN requires only 9.3 × 10${}^{-4}$ ms. Therefore, the proposed mechanism greatly suits the resource constrained sensors. The details of the comparison is shown in Figure 10.

## 6. Conclusions

In this paper, a channel characteristic aware privacy protection mechanism is proposed for WBAN, which is lightweight and only relies on channel characteristics to achieve autonomous node authentication and extracts real-time keys to encrypt the information. The analysis and experimental results demonstrate the advantages of the proposed mechanism. At the same time, this mechanism is proved to be more suitable for resource constrained WBANs. In the further work, we plan to optimize the fuzzy extraction method to further reduce the computation cost.

## Figures and Tables

**Table 1:** The meaning of notations.

Notation	Meaning
Y(d)	The received signal intensity of arbitrary distance *d*.
$H_{s}$	The transmission power of sensor node.
$G_{s}$	The antenna gain of sensor node.
$G_{c}$	The antenna gain of the coordinator.
*f*	The system loss factor.
λ	The wavelength of the wireless signal.
β	The path loss factor.
*X*	A normal random variable.
$\bar{\varepsilon^{2}}\left(Y_{s_{1}},Y_{s_{2}}\right)$	The mean square error between the RSS observations of $s_{1}$ and $s_{2}$.
$\rho_{\left(Y_{s1},Y_{s2}\right)}$	The correlation coefficient.
α	The wave factor.

**Table 2:** NIST test results for generating keys under different dimensions.

Dimensional	1	2	3	4	5	6	7	8
Frequency	0.48	0.21	0.35	0.12	0.68	0.68	0.31	0.025
Block frequency	0.99	0.53	0.58	0.48	0.48	0.91	0.87	0.24
Cumulative sums (Fwd)	0.39	0.27	0.14	0.35	0.18	0.18	0.68	0.43
Cumulative sums (Rev)	0.87	0.58	0.18	0.74	0.99	0.16	0.27	0.16
Runs	0.68	0.91	0.68	0.74	0.78	0.24	0.18	0.53
Longest runs of ones	0.96	0.4	0.87	0.74	0.27	0.74	0.96	0.017
FFT	0.28	0.1	0.23	0.21	0.31	0.17	0.07	0.00
Approximate entropy	0.53	0.78	0.12	0.35	0.91	0.24	0.35	0.41
Serial	0.21, 0.4	0.83, 0.48	0.63, 0.63	0.78, 0.79	0.21, 0.53	0.87, 0.53	0.03, 0.16	0.04, 0.63
